# Ocular antigen does not cause disease unless presented in the context of inflammation

**DOI:** 10.1038/s41598-017-14618-z

**Published:** 2017-10-27

**Authors:** Valentina Voigt, Matthew E. Wikstrom, Jelena M. Kezic, Iona S. Schuster, Peter Fleming, Kimmo Makinen, Stephen R. Daley, Christopher E. Andoniou, Mariapia A. Degli-Esposti, John V. Forrester

**Affiliations:** 1 Immunology and Virology Program, Centre for Ophthalmology and Visual Science, The University of Western Australia, Crawley, Western Australia Australia; 2 Centre for Experimental Immunology, Lions Eye Institute, Nedlands, Western Australia Australia; 3 Nestlé Research Center, Lausanne, Switzerland; 4 Infection and Immunity Program, Monash Biomedicine Discovery Institute and Department of Biochemistry and Molecular Biology, Monash University, Melbourne, Victoria Australia; 5 University of Aberdeen, Institute of Medical Sciences, Aberdeen, United Kingdom

## Abstract

Ocular antigens are sequestered behind the blood-retina barrier and the ocular environment protects ocular tissues from autoimmune attack. The signals required to activate autoreactive T cells and allow them to cause disease in the eye remain in part unclear. In particular, the consequences of peripheral presentation of ocular antigens are not fully understood. We examined peripheral expression and presentation of ocular neo-self-antigen in transgenic mice expressing hen egg lysozyme (HEL) under a retina-specific promoter. High levels of HEL were expressed in the eye compared to low expression throughout the lymphoid system. Adoptively transferred naïve HEL-specific CD4^+^ T cells proliferated in the eye draining lymph nodes, but did not induce uveitis. By contrast, systemic infection with a murine cytomegalovirus (MCMV) engineered to express HEL induced extensive proliferation of transferred naïve CD4^+^ T cells, and significant uveoretinitis. In this model, wild-type MCMV, lacking HEL, did not induce overt uveitis, suggesting that disease is mediated by antigen-specific peripherally activated CD4^+^ T cells that infiltrate the retina. Our results demonstrate that retinal antigen is presented to T cells in the periphery under physiological conditions. However, when the same antigen is presented during viral infection, antigen-specific T cells access the retina and autoimmune uveitis ensues.

## Introduction

Sight-threatening intraocular inflammation (uveitis) is the fourth most common cause of blindness, equivalent in frequency to that of diabetic retinopathy^1–3^, yet it is a relatively neglected disease. This is partly due to the large number of uveitis entities^4^ broadly grouped (a) anatomically, as anterior and intermediate/posterior; and (b) etiologically, as infectious or non-infectious^5^.

Infectious causes of uveitis account for around 50% of cases and are normally treated with antimicrobials (reviewed in^6^). The remaining 50% of cases are believed to be autoimmune, or at least immune-mediated, despite the varying presentations^6^. Experiments in rodents, particularly mice, have provided the strongest evidence for an autoimmune aetiology since uveitis can be induced by immunisation with defined peptides from highly conserved retinal proteins^7,8^. Indeed, the mouse model of experimental autoimmune uveitis (EAU) faithfully reflects human disease since its manifestations mirror the clinical signs of posterior uveitis, especially retinal vasculitis^9^ (for review, also see^6,10^). Studies of murine EAU have established that Th1 (IFN-γ-producing) and/or Th17 (IL-17-producing) CD4^+^ T cells^11–13^ are critical for the development of disease, whereby retina-specific T cells activated by immunisation are free to cross the blood-retina barrier due to upregulation of adhesion molecules and chemokine receptors^14–16^. A variety of other cell types (e.g. monocytes, neutrophils, and polyclonal T cells), recruited to the eye by cytokines and inflammatory mediators, also contribute to disease development (reviewed in^10^). Thus, a cascade of events produces autoimmune inflammation and tissue destruction in the eye following infiltration by activated CD4^+^ T cells.

Infectious agents, particularly viruses, have long been proposed as one of the environmental triggers of autoimmune disease^17–19^ including autoimmune uveitis^20–23^, though the mechanisms have not been characterised. Herpes simplex virus (HSV) 1 has been strongly associated with the development of stromal keratitis due to the similarities between the HSV UL6 protein and an unidentified corneal tissue antigen^24,25^. Similarly, molecular mimics for retinal S antigen have been identified in a variety of viruses and immunisation with these peptides induces autoimmune uveitis in rats^26,27^. Thus, there are instances when molecular mimicry may account for the development of autoimmunity in the eye. In contrast, the immunosuppressive environment of the eye appears to limit the potential for bystander activation^28^, although such activation cannot be ruled out^29^.

We have previously described a mouse model where EAU develops spontaneously, rather than in response to immunisation with ocular antigen and an adjuvant. Our model employs two transgenic systems; first, transgenic expression of a neo-antigen (hen egg lysozyme, HEL) in the retina under the control of the IRBP (interphotoreceptor retinoid-binding protein) promoter; second, 3A9 mice that express a HEL-specific T cell receptor on peripheral CD4^+^ T cells (HEL-TCR mice). When single transgenic (sTg-IRBP:HEL) mice are crossed with HEL-TCR mice, the double transgenic (dTg-IRBP:HEL) offspring develop EAU, with the first signs of disease seen at around 21 days of age^30^. By 5 weeks of age, all mice have developed disease, and by 6 weeks, most exhibit severe (grade 4) disease, both histologically and clinically^30^. At the peak of disease, there is severe vasculitis and granuloma formation in the retina, loss of photoreceptors, and extensive infiltration by macrophages and T cells – all features that replicate presumptive autoimmune uveoretinitis in humans (Forrester, personal unpublished data).

In this report, we utilised adoptive transfer of naïve T cells from HEL-TCR mice to examine presentation of HEL in the periphery of two strains of sTg-IRBP:HEL mice that differ in their expression of HEL (sTg-IRBP:HEL^lo^ versus sTg-IRBP:HEL^hi^). Transient T cell proliferation was noted in both strains, but uveoretinitis did not develop. In contrast, systemic infection with murine cytomegalovirus (MCMV) expressing an epitope of HEL stimulated extensive proliferation of naïve 3A9 CD4^+^ T cells and induced uveoretinitis, where the severity of disease was dependent on the level of HEL expression in the retina. In the absence of HEL epitope expression in this model, MCMV infection did not induce overt uveoretinitis. These results confirm that the mechanisms that regulate autoimmunity in peripheral organs, by limiting T cell activation and proliferation, apply equally to the eye. However, presentation of an ocular antigen during infection with a virus expressing that antigen, led to disease suggesting that the context of peripheral antigen presentation predicates the risk of autoimmune inflammation.

## Results

### IRBP:HEL^lo^ mice develop less severe ocular inflammation than IRBP:HEL^hi^ mice when crossed with HEL-TCR transgenic mice

**Figure 1:**
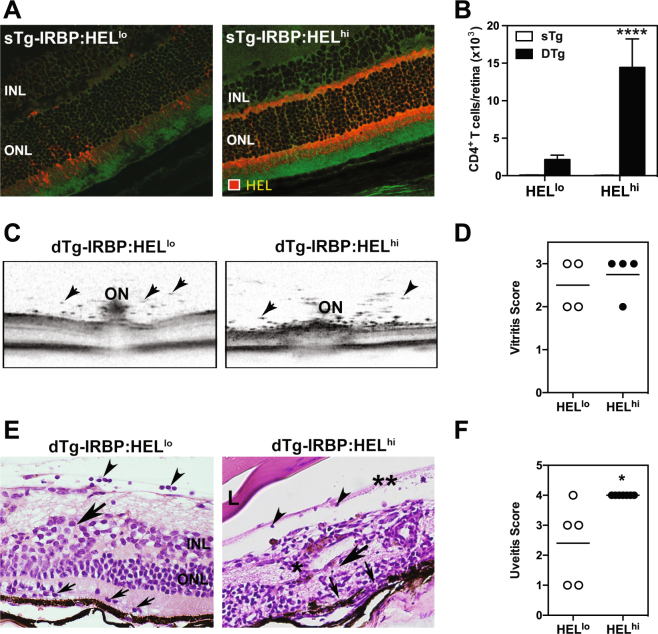
sTg-IRBP:HEL mice expressing different levels of HEL in the retina are both susceptible to autoimmune uveitis. (**A**) HEL expression in the retinas of adult sTg-IRBP:HEL^lo^ versus sTg-IRBP:HEL^hi^ mice. Red = HEL, green = background autofluorescence. INL; inner nuclear layer, ONL; outer nuclear layer. (**B**) The number of CD4^+^ T cells present in the retinas of adult IRBP:HEL^lo^ and IRBP:HEL^hi^ mice after the onset of disease (5–6 weeks of age) was determined by flow cytometry for single trangenic (sTg short for sTg-IRBP:HEL) and double transgenic (dTg short for dTg-IRBP:HEL) mice. Results are for pooled samples of both retinas from at least 5 (5–10) individual mice for each group. ****p < 0.0001 (dTg-IRBP:HEL^lo^ vs dTg-IRBP:HEL^hi^). (**C**) OCT images of central retina in the region of the optic nerve (ON) in dTg-IRBP:HEL^lo^ and dTg-IRBP:HEL^hi^ mice. Vitritis is demonstrated as discrete spots indicating cellular aggregates (arrowheads). (**D**) Vitritis scores as assessed by OCT. (**E**) Representative H&E stained histological sections of retinas from dTg-IRBP:HEL^lo^ and dTg-IRBP:HEL^hi^ mice. dTg-IRBP:HEL^lo^ mice show mild to moderate signs of inflammation with infiltration of photoreceptor outer segment layers with mononuclear cells (small arrows), vitritis (arrow heads) and disruption of the inner and outer nuclear layers (large arrow). dTg-IRBP:HEL^hi^ mice show signs of vitiritis (arrow heads), retinal necrosis (large arrow), complete absence of photoreceptors (small arrows) and perivascular retinal granuloma (*). L = lens. **Shows condensed posterior vitreous detachment. (**F**) Uveitis scores in dTg-IRBP:HEL^lo^ and dTg-IRBP:HEL^hi^ mice. *p = 0.0101.

We previously demonstrated that IRBP:HEL^hi^ mice spontaneously develop autoimmune uveitis (EAU) when crossed with HEL-TCR transgenic mice^30^. A second line of single transgenic (sTg)-IRBP:HEL mice was generated, IRBP:HEL^lo^ mice, which expressed lower levels of HEL in the retina, confined to discrete patches of retinal photoreceptor, compared to the uniform distribution and high level of HEL photoreceptor expression observed in sTg-IRBP:HEL^hi^ mice (Fig 1A). HEL expression in sTg-IRBP:HEL^hi^ is associated with some degree of age-related retinal thinning due to the presence of this transgene and reduced levels of IRBP, which is known to be essential for retinal health in adult mice (Forrester, unpublished data). EAU occurs in 100% of dTg-IRBP:HEL^hi^ mice derived by crossing sTg-IRBP:HEL^hi^ mice with HEL-TCR mice, despite the retinal thinning, indicating that in this mouse strain the level of retinal HEL antigen expression is sufficient to consistently induce EAU. Accordingly, the dTg-IRBP:HEL mouse is a useful model to investigate mechanisms of immunological tolerance in the retina.

We first wished to compare the clinico-pathological features of EAU in sTg-IRBP:HEL^hi^ vs sTg-IRBP:HEL^lo^ mice when they were crossed with the HEL-TCR mice. A comparison of CD4^+^ T cells by flow cytometry in the retina after the onset of EAU (5–6 weeks of age) revealed fewer CD4^+^ T cells in dTg-IRBP:HEL^lo^ mice when compared to dTg-IRBP:HEL^hi^ mice (Fig. 1B; p < 0.0001). Furthermore, intraocular inflammation in dTg-IRBP:HEL^lo^ mice was less severe than that seen for dTg-IRBP:HEL^hi^ mice, as examined by optical coherence tomography (OCT) (Fig. 1C,D) and histology (Fig. 1E,F; p = 0.0101). Infiltration of the retina and damage to the outer photoreceptor cell layer appeared to be confined to discrete patches in dTg-IRBP:HEL^lo^ mice when compared to the widespread infiltration and photoreceptor damage observed in dTg-IRBP:HEL^hi^ mice (Fig. 1E). Taken together, these results demonstrate that the level of HEL expression in the retina determined the severity of EAU in dTg-IRBP:HEL mice.

### HEL is expressed in the periphery of sTg-IRBP:HEL mice

**Figure 2:**
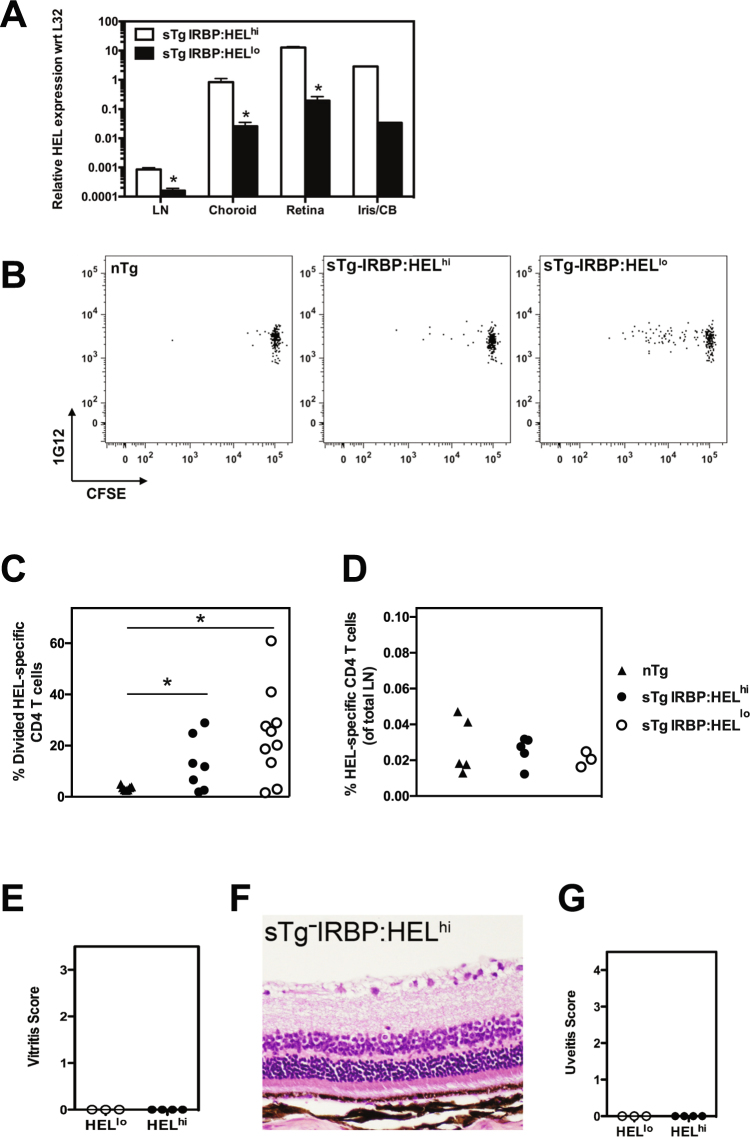
HEL is expressed in the periphery of sTg-IRBP:HEL mice. (**A**) Lymph nodes (LN) and eyes were collected from sTg-IRBP:HEL^hi^ and sTg-IRBP:HEL^lo^ mice. The eyes were dissected to separate the iris/ciliary body (Iris/CB) from the retina and choroid/RPE (Choroid). The tissues were lysed and homogenised for mRNA preparation. After cDNA was prepared, the relative abundance of HEL was determined by RT-PCR with respect to L32, a housekeeping gene. n = 3–4 mice for all tissues except iris, which represents the result for the irises pooled from 4 mice. *p < 0.05. (**B**–**D**) Adult non-Tg, sTg-IRBP:HEL^lo^ and sTg-IRBP:HEL^hi^ mice were administered CFSE-labelled lymph node cells from HEL-TCR donors. Four days (**B**,**C**) or 30 days (**D**) later, peripheral lymph nodes were collected and the dilution of CFSE was determined for donor CD4^+^1G12^+^ (HEL-specific) T cells. (**B**) Representative CFSE profiles for CD4^+^1G12^+^ T cells 4 days after adoptive transfer. (**C**) Frequency of HEL-specific CD4^+^ T cells that had divided at least once 4 days after adoptive transfer. *p < 0.05. (**D**) Frequency of HEL-specific CD4^+^ T cells in the lymph nodes 30 days after adoptive transfer. Results are collective from 2–3 independent experiments. (**E–G**) Adult sTg-IRBP:HEL^lo^ and sTg-IRBP:HEL^hi^ mice were administered T cells from HEL-TCR donors. (**E**) Eyes were examined and graded for evidence of vitritis by OCT 15 days after transfer. (**F**,**G**) Histological analysis was carried out 29 days post transfer. (**F**) Representative tissue section from a sTg-IRBP:HEL^hi^ mouse. (**G**) Uveitis was scored for both groups of mice. Results are shown for at least 3 mice/group.

We have previously shown that low levels of HEL are expressed in the thymus, while high levels are expressed in the retina of IRBP:HEL^hi^ mice^30^. To determine whether HEL was expressed in the periphery, we compared the level of HEL expression in the eye of sTg-IRBP:HEL^hi^ and sTg-IRBP:HEL^lo^ mice with that in peripheral lymph nodes using RT-PCR. HEL mRNA was detected at high levels in the choroid/RPE and the iris/ciliary body (CB), as well as the retina, while much lower levels were detected in the peripheral lymph nodes (LN) of both sTg-IRBP:HEL mouse strains (Fig. 2A). As expected, HEL expression was consistently higher in sTg-IRBP:HEL^hi^ mice, ranging from a difference of 4-fold for the lymph nodes to almost 100-fold for the iris when compared to sTg-IRBP:HEL^lo^ mice (Fig. 2A).

### HEL is presented in the lymph nodes of sTg-IRBP:HEL mice but EAU does not develop

Adoptive transfer of congenic CD45.1^+^ CFSE-labelled 3A9 T cells (herein referred to as HEL-specific CD4^+^ T cells) from HEL-TCR mice was used to assay for the presentation of HEL in the peripheral lymph nodes of sTg-IRBP:HEL^lo^ and sTg-IRBP:HEL^hi^ mice (expressing CD45.2^+^). In this assay, the extent of CFSE dilution is directly proportional to the strength and/or frequency of antigen presentation^31^. We found that a small proportion of HEL-specific CD4^+^ T cells (identified by the clonotype-specific MAb 1G12) proliferated in the lymph nodes 4 days after adoptive transfer in both strains of sTg-IRBP:HEL mice, while no CFSE dilution was observed in non-transgenic (nTg) control mice (Fig. 2B). The extent of CFSE dilution was higher in sTg-IRBP:HEL^lo^ (p = 0.0172) and sTg-IRBP:HEL^hi^ mice (p = 0.0315) when compared to nTg mice, but there was no significant difference in proliferation between the sTg mice (Fig. 2C). Day 4 proved to be the peak of proliferation, with no change in the proportion of divided cells or the overall frequency of CD4^+^1G12^+^ cells at later time points (data not shown). One month after transfer, the majority of donor T cells remained undivided and persisted in the lymph nodes at the same frequency observed for nTg controls (Fig. 2D).

Examination of the eyes of recipient mice by OCT revealed no evidence of vitritis at either early (Fig. 2E; day 15 post adoptive transfer) or late time points (data not shown; day 29 post adoptive transfer). Histological analysis at day 29 post adoptive transfer of HEL-specific CD4^+^ T cells confirmed the OCT observations, with no sign of inflammation in either the vitreous or the retina of sTg-IRBP:HEL^lo^ or sTg-IRBP:HEL^hi^ (Fig. 2F,G) mice. Thus, peripheral presentation of HEL was sufficient to stimulate transient proliferation of a small proportion of naïve specific T cells, but was not sufficient to induce EAU.

### HEL expression by a virus induces EAU

The data shown above demonstrated that retinal neo-self antigen (HEL) was recognised in the periphery by antigen-specific T cells, but did not induce disease. Previous studies with neo-self-antigen expression in the pancreas established that autoimmunity could be induced by a shared epitope expressed by a viral vector^32,33^. Given that the retina is considered to be an “immune privileged” tissue, courtesy in part of the blood retinal barrier^34,35^, we tested whether this notion applies equally to ocular antigens. To examine this we engineered a mouse cytomegalovirus (MCMV) expressing the HEL epitope recognised by HEL-specific CD4^+^ T cells (see 2.3 Methods), and infected sTg-IRBP:HEL (CD45.2^+^) mice one day after adoptive transfer of HEL-specific CD4^+^ T cells from HEL-TCR (CD45.1^+^) mice.

**Figure 3:**
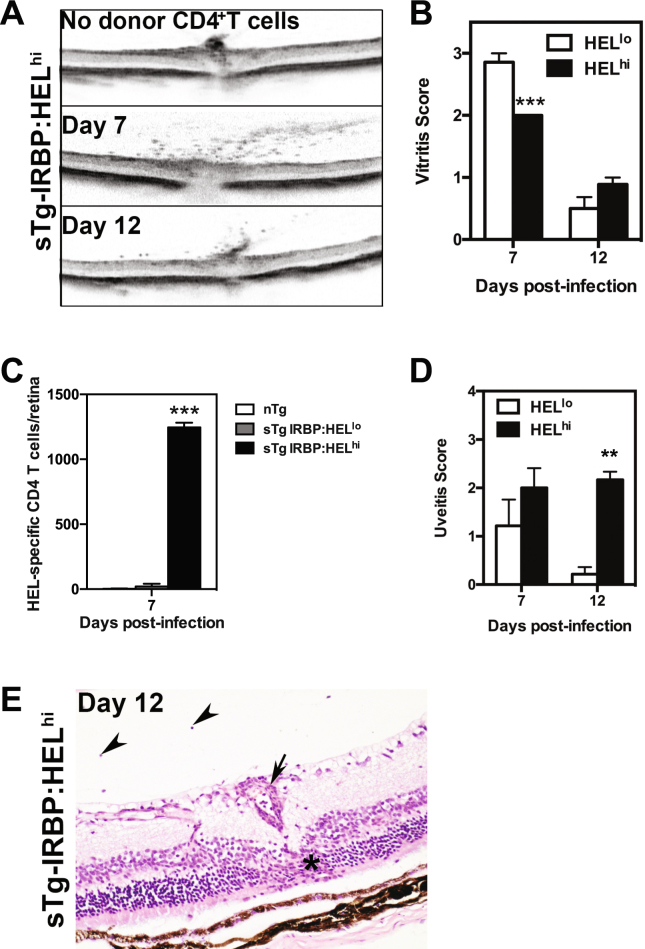
MCMV-HEL induces uveitis in sTg-IRBP:HEL mice after adoptive transfer of HEL-specific CD4^+^ T cells. Adult sTg-IRBP:HEL^lo^ and sTg-IRBP:HEL^hi^ mice (CD45.2^+^) were administered T cells from HEL-TCR (CD45.1^+^) donors. One day later, mice were infected i.p. with 5 × 10^4^ pfu of MCMV-HEL, a recombinant MCMV expressing the HEL epitope recognised by HEL-specific CD4^+^ T cells. (**A**,**B**) Eyes were examined 7 and 12 days post-infection (p.i.) by OCT for evidence of vitritis. (**A**) Representative images from sTg-IRBP:HEL^hi^ mice at days 7 and 12 p.i. Note the absence of inflammatory cells in mice that were infected but had not received HEL-specific CD4^+^ T cells. sTg-IRBP:HEL^hi^ mice display some degree of retinal thinning which is due to the presence of the HEL transgene in the photoreceptor membrane^30^ and associated reduced levels of IRBP (see also Fig. 1 and text). (**B**) The severity of vitritis was scored for both groups of mice at each timepoint. Results are shown for at least 6 mice/group. ***p = 0.0009. (**C**) Eyes were collected from sTg-IRBP:HEL^lo^ and sTg-IRBP:HEL^hi^ mice 7 days p.i. and the number of donor HEL-specific CD4^+^ T cells in the retina was determined by flow cytometry. Results are from 3 mice/group. ***p < 0.0001. (**D**) Eyes were collected 7 and 12 days p.i. for histological analysis and the severity of uveitis scored for each group. Results are for 3–7 mice/group. **p = 0.0083 (**E**) Representative image from sTg-IRBP:HEL^hi^ mice at day 12 p.i. showing retinal vasculitis (small arrow), retinal granuloma in photoreceptor layer (*) and vitritis (arrow heads).

Examination of the eyes of sTg-IRBP:HEL^hi^ mice by OCT demonstrated that infection with MCMV-HEL induced vitritis which developed 7 days post-infection and declined in severity by day 12 (Fig. 3A,B). Vitritis was also observed with a similar pattern, albeit milder (p = 0.0009 at day 7 post-infection), in sTg-IRBP:HEL^lo^ mice (Fig. 3B). This inflammation was dependent on the adoptive transfer of HEL-specific CD4^+^ T cells, since vitritis was not detected in control sTg-IRBP:HEL^lo^ (data not shown) or sTg-IRBP:HEL^hi^ (Fig. 3A) mice that did not receive HEL-specific CD4^+^ T cells prior to infection with MCMV-HEL. At the peak of vitritis (day 7 post-infection), there was a marked difference in the number of antigen-specific CD4^+^ T cells infiltrating the retinas of sTg-IRBP:HEL^lo^ and sTg-IRBP:HEL^hi^ mice, where the latter contained ~50-fold more HEL-specific CD4^+^ T cells (Fig. 3C; p < 0.0001). Histological examination of ocular tissue revealed less severe uveitis scores in sTg-IRBP:HEL^lo^ mice when compared to sTg-IRBP:HEL^hi^ mice at both time points, with a significant difference at day 12 post-infection (Fig. 3D; p = 0.0083). sTg-IRBP:HEL^hi^ mice displayed evidence of vasculitis, patchy granuloma formation, and cellular infiltration of the retina, along with damage to the outer nuclear layer and photoreceptor cell layer at day 12 (Fig. 3E). The severity of ocular inflammation in sTgIRBP:HEL^hi^ mice was similar to, but not as severe or as long lasting as that seen for dTg-IRBP:HEL mice (Fig. 1).

### CD4^+^ T cells proliferate in the periphery prior to infiltrating the retina

**Figure 4:**
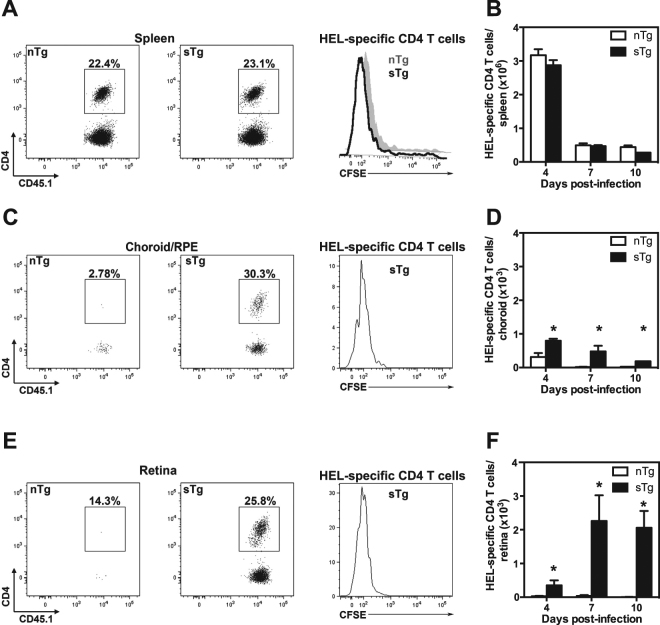
CD4^+^ T cells proliferate and infiltrate the retina following MCMV-HEL infection. Non-Tg (nTg) and sTg-IRBP:HEL^hi^ (CD45.2^+^) mice were administered CFSE-labelled lymph node cells from HEL-TCR donors (CD45.1^+^). One day later, the mice were infected i.p. with 5 × 10^4^ pfu recombinant MCMV expressing the HEL epitope recognised by HEL-specific CD4^+^ T cells. The spleen and eyes were collected at 4 and 7 days p.i. and dissected for detection of donor CD45.1^+^ CD4^+^ T cells by flow cytometry. Cells were gated on CD45.1^+^ and CD45.2^−^. The percentage of CD4^+^ cells of the donor population (CD45.1^+^/CD45.2^−^) is shown. (**A**) Donor HEL-specific CD4^+^ T cells identified in the spleen on day 7 p.i. and the dilution of CFSE compared for each group of recipients. (**B**) The number of donor HEL-specific CD4^+^ T cells was calculated for the spleen at the indicated time points. (**C**) Donor HEL-specific CD4^+^ T cells were detected in the choroid/RPE on day 7 p.i. and the dilution of CFSE compared for each group of recipients. There were negligible HEL-specific CD4^+^ T cells in the choroid/RPE of nTg mice, thus these cells cannot be seen in the CFSE profile. (**D**) The number of donor HEL-specific CD4^+^ T cells was calculated for the choroid/RPE at the indicated timepoints (days post infection *p = 0.045; nTg vs sTg *p = 0.02). (**E**) Donor HEL-specific CD4^+^ T cells were detected in the retina on day 7 p.i. and the dilution of CFSE compared for each group of recipients. There were negligible HEL-specific CD4^+^ cells in the retina of nTg mice, thus these cells cannot be seen in the CFSE profile. (**F**) The number of donor HEL-specific CD4^+^ T cells was calculated for the retina at the indicated timepoints (days post infection not significant; nTg vs sTg *p = 0.0007). Results are shown for 3–6 mice/group; data for the choroid/RPE were obtained from pooled samples for each group. Results are collective from 1–2 independent experiments.

We used CFSE-labelling to track the proliferation of HEL-specific CD4^+^ T cells (CD45.1^+^) in sTg-IRBP:HEL^hi^ mice following MCMV-HEL infection. The expression of HEL had no impact on the extent of splenic T cell proliferation, since similar CFSE profiles and HEL-specific CD4^+^ T cell numbers were observed in non-transgenic (nTg) control mice and sTg-IRBP:HEL^hi^ mice after infection with MCMV-HEL (Fig. 4 A,B). HEL-specific CD4^+^ T cells were detected in the pooled samples of choroid/RPE of both sTg-IRBP:HEL^hi^ mice and nTg controls on day 4 (Fig. 4D) and day 7 (Fig. 4C,D); however, the extent of infiltration was greater and longer lasting in sTg-IRBP:HEL recipients than in nTg mice (Fig. 4D; days post infection p = 0.045; nTg vs sTg p = 0.02).

In contrast, large numbers of proliferating HEL-specific CD4^+^ T cells were detected in the retinas of sTg-IRBP:HEL^hi^ mice on day 4 (Fig. 4F), peaking 7–10 days post-infection (Fig.  4E,F; days post infection not significant; nTg vs sTg, p = 0.0007). Only very small numbers of HEL-specific CD4^+^ T cells were detected in the retinas of nTg controls (Fig. 4E,F), demonstrating that while large numbers of activated CD4^+^ T cells were generated in the periphery in response to MCMV-HEL, infiltration of the retina was dependent on the presence of antigen in this tissue.

**Figure 5:**
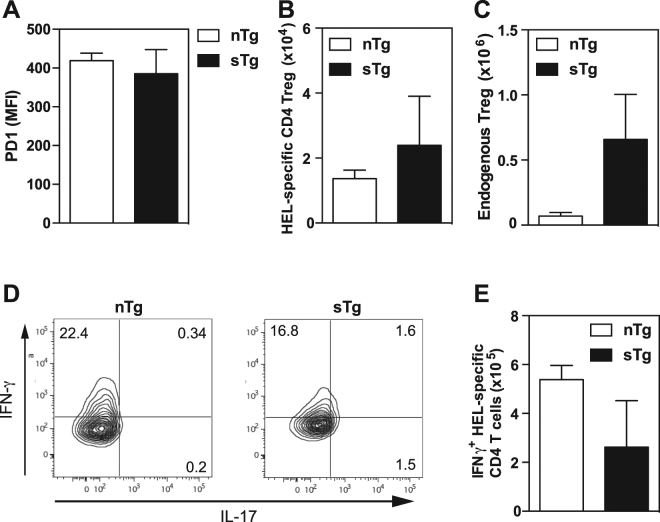
Endogenous expression of HEL does not alter activation of HEL-specific CD4^+^ T cells. HEL-specific T cells were transferred into non-Tg or sTg-IRBP:HEL^hi^ mice and 24 h later mice were infected with 5 × 10^4^ pfu MCMV-HEL. At day 4 p.i. spleens were removed and single cell preparations prepared. FACS analysis was performed to determine (**A**) expression of PD1 by HEL-specific CD4^+^ T cells (MFI: mean fluorescence intensity), (**B**) number of HEL-specific CD4^+^ T cells with a Treg phenotype and (**C**) number of endogenous Treg cells. (**D**) Representative FACS plots assessing production of IFN-γ and IL-17 by HEL-specific CD4^+^ T cells at day 4 p.i. (**E**) Number of HEL-specific CD4^+^ T cells producing IFN-γ 4 days after MCMV-HEL infection. Results are shown for 3 mice/group and are representative of 1–2 independent experiments.

Since regulatory T cells (Treg) and the PD-1 pathway are critical in the development of autoimmunity including EAU^36–38^, we examined the expression of PD-1 and the Treg marker Foxp3 in HEL-specific CD4^+^ T cells after MCMV-HEL infection. At day 4 post infection, HEL-specific CD4^+^ T cells from sTg-IRBP:HEL mice and nTg controls displayed comparable levels of PD-1 (Fig. 5A) and numbers of HEL-specific CD4^+^ Foxp3^+^ Treg were equivalent in these mice (Fig. 5B). Recipient-derived Treg cells could potentially affect the expansion of the HEL-specific CD4^+^ T cells; however, the numbers of endogenous splenic Foxp3^+^ Treg cells were higher in sTg-IRBP:HEL^hi^ mice than in nTg control mice (Fig. 5C). These data suggest that, at least in the periphery, HEL-specific CD4^+^ T cells are as efficiently activated in the presence or absence of antigen and that they do not contain a significant antigen-specific Treg subset. Furthermore, after MCMV-HEL infection the higher endogenous Treg responses observed in sTg-IRBP:HEL^hi^ mice, indicate that at least in the periphery, Treg cells do not contribute to protection from EAU in this model.

Both Th1 (IFN-γ) and/or Th17 (IL-17) cells have been associated in the pathogenesis of EAU^12^, hence we investigated the cytokine profile of the HEL-specific CD4^+^ T cells activated in our infection model. On day 4 post infection with MCMV-HEL, no IL-17 production was found in HEL-specific CD4^+^ T cells in either nTg control mice or sTg-IRBP:HEL^hi^ mice (Fig. 5D), but these CD4^+^ T cells produced IFN-γ (Fig. 5D,E). The number of HEL-specific CD4^+^ T cells producing IFN-γ was not increased in sTg-IRBP:HEL^hi^ mice compared to nTg controls (Fig. 5E), indicating that like proliferation, activation of autoreactive CD4^+^ T cells in the periphery was equivalent.

### MCMV does not induce bystander activation of HEL-specific CD4^+^ T cells or EAU

**Figure 6:**
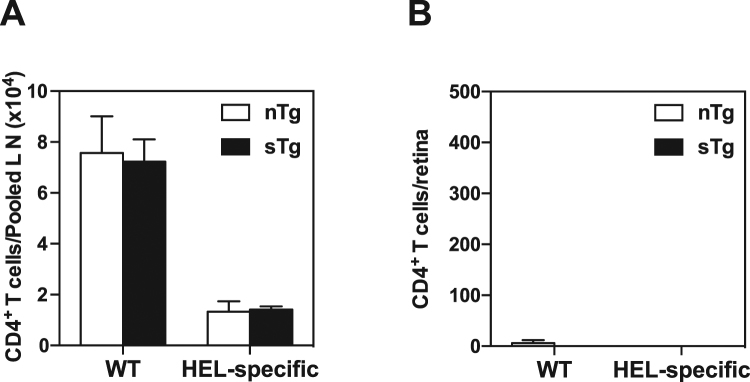
MCMV without a HEL epitope does not induce activation of HEL-specific CD4^+^ T cells or infiltration of the retina. Non-Tg (nTg) and sTg-IRBP:HEL^hi^ mice were administered CFSE-labelled CD4^+^ cells as a 1:1 mixture of HEL-specific CD4^+^ T cells (CD45.1^+^) and non-transgenic (WT) cells (CD45.2^+^). One day later, the mice were infected i.p. with 5 × 10^4^ pfu MCMV. The lymph nodes and retinas were collected from each recipient 6 days later and prepared for detection and enumeration of HEL-specific CD4^+^ donor T cells by flow cytometry. (**A**) The number of HEL-specific CD4^+^ donor T cells was determined for the lymph nodes (LN) from each group of recipients for each donor population. (**B**) The number of donor CD4^+^ T cells was determined for the retinas from each group of recipients for each donor population. Few donor cells were detected in the retinas of either group of recipients. Results are shown for 3 mice/group. Results are representative of two independent experiments.

Viral infection is well known to affect antigen presentation by professional antigen-presenting cells, increasing the potential for enhanced presentation of self-antigen^17,19^. We examined this possibility in our model by transferring a 1:1 mix of naïve HEL-specific CD4^+^ T cells (CD45.1^+^) and non-transgenic CD4^+^ cells (CD45.2^+^, wild-type, WT) into sTg-IRBP:HEL^hi^ mice and nTg controls. The two populations were distinguished by differential expression of CD45.1 and CD45.2 and dilution of CFSE was examined 7 days after infection with wild-type MCMV (i.e. lacking HEL expression). Very few HEL-specific CD4^+^ T cells had proliferated in the lymph nodes of sTg-IRBP:HEL^hi^ mice by day 7 post-infection compared to multiple rounds of proliferation noted in a proportion of the non-transgenic WT donor cells (data not shown). Furthermore, the expansion of the non-transgenic WT donor CD4^+^ T cells was identical in both sTg-IRBP:HEL^hi^ and nTg recipients, while the number of HEL-specific CD4^+^ T cells, also identical for both groups of recipients, was much lower (Fig. 6A). A small number of non-transgenic WT CD4^+^ T cells were detected in the retina on day 7 post-infection, while few if any HEL-specific CD4^+^ donor T cells were present (Fig. 6B). At the same time, there was no evidence of overt ocular inflammation or pathology (data not shown). Thus, infection with MCMV lacking a shared epitope with the retinal neo-antigen HEL did not enhance the presentation of HEL in sTg-IRBP:HEL^hi^ mice, nor did it induce overt uveoretinitis through bystander effects.

## Discussion

Expression of tissue-specific antigens in the thymus is crucial for central tolerance^39^. Ectopic expression of these antigens also occurs in other peripheral lymphoid tissues, and there is evidence that presentation by stromal cell types is a crucial component of peripheral tolerance^40–42^. In the present study, we utilised a system whereby transgenic expression of a neo-antigen (hen egg lysozyme, HEL) in the retina is under the control of the IRBP promoter. When sTg-IRBP:HEL^lo^ and sTg-IRBP:HEL^hi^ mice, which differ in their expression of HEL, are crossed with HEL-TCR transgenic mice, double transgenic offspring spontaneously develop EAU, suggesting that the mechanisms that induce peripheral tolerance can be overwhelmed when there is endogenous overproduction of antigen-specific T cells. This notion is supported by recent experiments with transgenic mice expressing different levels of an IRBP-specific TCR, where EAU developed spontaneously in lines with a high frequency of antigen-specific CD4^+^ T cells (>50% of the CD4^+^ T cell compartment), but not in those with a low frequency (5% of the CD4^+^ T cell compartment)^43^.

Studies in mice have clearly demonstrated that activation of T cells in the periphery by immunisation with ocular proteins in adjuvant is sufficient to induce EAU^7^. Similarly, transgenic mice expressing neo-self-antigens in the eye are susceptible to EAU as long as suitably primed T cell effectors are administered or generated *in vivo* ^44–48^. T cells progressively differentiate to acquire effector functions as they proliferate^49^, along with the ability to infiltrate non-lymphoid tissues^31^, which in turn is driven by the strength of antigen presentation^50^. Adjuvants, TLR ligands, and viral pathogens are well known for enhancing antigen presentation, and thus their importance to the induction of EAU is most likely to lie with the generation of suitably primed T cell effectors. Our experiments provide novel evidence that a shared viral epitope is capable of inducing autoimmune uveitis. Furthermore, we demonstrate that infiltration of the retina is antigen-specific, at least during the acute phase of disease development. Shared epitopes between pathogens, commensals and self-antigens is recognised as a possible route to autoimmune disease. Recent studies indicate that peripheral activation of retinal antigen specific T cells takes place in the gut and is mediated by commensal antigens^51^. Whether this is due to a shared epitope between the retina and commensal organism or is due to TCR degeneracy is not clear^52,53^. However, epitope redundancies between pathogen and self-antigen may not be sufficient to induce disease.

Interestingly, although several transgenic models of EAU have been utilised (see^7^), not all are equally susceptible to disease, in part reflecting the different antigens expressed, as well as potential strain differences in levels of ocular immune privilege. For instance, in the beta-galactosidase model used by Gregerson’s group, EAU develops following adoptive transfer of antigen specific T cells only if the mice are lymphopenic^44^. This work highlighted the importance of peripheral T regulatory (Treg) cells in determining the likelihood (or not) of EAU development^36,44^. It is therefore likely that the balance of Treg versus T effectors sets the threshold of risk for development of spontaneous EAU.

In the present study, sTg-IRBP:HEL^lo^ and sTg-IRBP:HEL^hi^ mice did not develop EAU following adoptive transfer of naïve HEL-specific CD4^+^ T cells (3A9 T cells) alone, but the combination of naive HEL-specific CD4^+^ T cells with MCMV-HEL infection 1 day post adoptive transfer resulted in intraocular inflammation. In this setting, Th1 (IFN-γ) and/or Th17 (IL17) cells would be expected to be involved in the pathogenesis of EAU. Indeed, we did note IFN-γ production by proliferating HEL-specific CD4^+^ T cells in lymphoid organs (spleen, cervical lymph nodes) in response to MCMV-HEL infection in both sTg-IRBP:HEL mice that had received HEL-specific CD4^+^ T cells. Under these conditions, the majority of antigen-specific CD4^+^ T cells proliferated extensively in the spleen and lymph nodes within 4 days of infection and produced IFN-γ. CD4^+^ T cells that infiltrated the retina had also undergone multiple cell divisions. In this study we did not examine cytokine production by HEL-specific CD4^+^ T cells that had infiltrated the retina, but expect that they will produce IFN-γ as they do in the periphery. This notion is consistent with the finding that EAU induced by antigen-pulsed dendritic cells (DC) is IFN-γ dependent. In the DC-mediated EAU model, retinal antigen is presented by DC activated with lipopolysaccharide and anti-CD40^54^, a condition that is induced by the viral infection used in our studies.

Uveitis was more severe in sTg-IRBP:HEL^hi^ mice when compared to sTg-IRBP:HEL^lo^ mice, with the extent of infiltration and the severity of tissue pathology correlating with HEL expression in the eye. This has previously been reported for other tissue-specific antigens^55,56^. Such an association strongly suggests that local presentation has an important role in the disease process. Dendritic cells and macrophages have been shown to reside in the uveal tract^57,58^ and in the peripheral retina^59^; however, local presentation by these cells has yet to be characterised. Studies on experimental autoimmune encephalomyelitis have provided evidence that T cells infiltrating the central nervous system must be activated by antigen presenting cells which may have preceded the T cells across the blood-brain barrier in order to induce disease^60,61^. Thus, it seems likely that T cells crossing the blood-retina barrier must also survey local antigen-presenting cells prior to infiltrating the retina to induce EAU, though the site at which this occurs remains to be determined.

Another critical requirement for the development of EAU is a population of antigen-specific T cells in the periphery. We found that a single dose of MCMV-HEL could not induce EAU in sTg-IRBP:HEL mice in the absence of HEL-specific CD4^+^ T cells, presumably due to deletion of HEL-specific T cells in the thymus (data not shown). Previous studies have shown that negative selection is crucial for tolerance of ocular antigens^47,62–64^ and indeed, we have observed extensive negative selection of HEL-specific CD4^+^ T cells in the thymi of dTg mice^30^. It remains to be determined how many antigen-specific T cells are required in the periphery for the development of EAU, though this is likely to depend upon the strength of the antigenic stimulus available. Thus, the risk of autoimmunity developing in response to tissue-specific antigens remains negligible until there is over-production of antigen-specific T cells (eg. as a consequence of a defect in negative selection). In contrast, an epitope that is shared by a virus poses a much greater risk since it is likely to provide a stronger antigenic stimulus for naïve T cells and at a lower T cell precursor frequency. Whilst the aim of the present study was to determine the signals required to prime autoreactive CD4^+^ T cells and to define their localisation to the retina, these studies also showed that regulatory T cells are unlikely to explain the differences in susceptibility to EAU noted in our viral infection model. Our ongoing studies are examining the role of Treg cells at later stages of the disease and specifically in the eye as these cells may be relevant to the resolution of uveitis, as described in other models^37^ and may be generated in the retina independently of circulating Treg cells^36^.

We found no evidence for bystander activation by MCMV by directly examining peripheral activation of HEL-specific CD4^+^ T cells in the lymph nodes of IRBP:HEL mice. MCMV did not enhance T cell proliferation stimulated by self-presentation of HEL nor did it induce infiltration of the eye by HEL-specific CD4^+^ T cells. Therefore, MCMV infection per se had no effect on HEL presentation, or the integrity of the blood-retina barrier. In contrast, small numbers of WT T cells were detected in the choroid, regardless of HEL expression, and the majority were CD8^+^ (unpublished data), which is consistent with local infection. Taken together, these data indicate that non-specific activation and the effects of local inflammation do not contribute to the development of EAU in our model.

In summary, we have examined the development of autoimmunity against a retinal antigen and found that: first, the severity of disease is driven by the level of protein expression in the retina; second, autoreactive CD4^+^ T cell infiltration of the retina is antigen-specific, although non-HEL specific T cells, as well as myeloid cells may also gain access to the retina; third, peripheral presentation of a tissue-specific antigen is capable only of stimulating weak proliferation of naïve CD4^+^ T cells and fourth, the development of EAU depends upon strong proliferation of antigen-specific T cells in the periphery. Our understanding of the mechanisms that enforce peripheral tolerance is still rudimentary, and the events that lead to the induction of spontaneous autoimmune disease are yet to be determined. The results presented here argue that peripheral activation of autoreactive CD4^+^ T cells is critical for the induction of disease, while local presentation propagates tissue pathology.

## Methods

### Animals

Single transgenic sTg-IRBP:HEL^hi^ mice that express high levels of HEL under the IRBP promoter have been described previously^30^. A second strain of mice, sTg-IRBP:HEL^lo^ mice, were generated at the same time that express lower levels of HEL in the retina. HEL-TCR transgenic mice recognise the epitope of HEL defined by residues 46–61 in the context of I-A^k^ ^65^. HEL-TCR mice expressing CD45.1 were provided by the Australian National University, Canberra, Australia. Double transgenic mice were generated by crossing HEL-TCR (CD45.1) mice with sTg-IRBP:HEL^hi^ and sTg-IRBP:HEL^lo^ mice (CD45.2) (dTg-IRBP:HEL). All mice were bred at the Animal Resources Centre (Perth, Australia) and housed in specific pathogen free conditions at the Animal Services Facility of UWA. All animal experimentation was performed with the approval of the UWA Ethics and Experimentation Committees, according to the guidelines of the National Health and Medical Research Council of Australia.

### Adoptive transfer of T cells

HEL-specific CD4^+^ T cells (3A9 T cells) were prepared for adoptive transfer from the lymph nodes of HEL-TCR (CD45.1) transgenic mice. When required, cells were labeled with CFSE (Invitrogen, USA) at a final concentration of 5 μM prior to adoptive transfer. 5–10 × 10^6^ 3A9 lymph node cells were re-suspended in 200 μl PBS and injected i.v. via the tail vein. Donor cells were identified on the basis of CD45.1 expression.

### Virus infection

MCMV (K181-Perth strain) expressing HEL residues 46–61 (MCMV-HEL) was created by inserting the coding sequence for NTDGSTDYGILQINSRWWCN into virus at the end of the ie1 gene. Mice were inoculated with salivary gland propagated stocks of WT MCMV (K181-Perth strain) or MCMV-HEL at a dose of 5 × 10^4^ plaque forming units/mouse i.p. Viruses were diluted in PBS (Gibco, USA) supplemented with 0.5% v/v FCS (Gibco, USA).

### RT-PCR

Message RNA was prepared after ocular and lymphoid tissues were lysed and homogenised using an Ambion PureLink RNA Mini kit according to the manufacturer’s instructions (Life Technologies, USA). Complimentary DNA was prepared by standard techniques and assayed for HEL specific sequences using RT-PCR. Results were normalised against L32, a housekeeping gene. The assay was performed on serially diluted samples and the melt curve of each examined for spurious products. Non-transgenic mice did not yield any results with the primers used.

### Optical coherence tomography

Mice were anesthetised systemically by intraperitoneal injection using a mixture of 20 mg/ml ketamine and 2 mg/ml xylazine (Troy Laboratories, Australia). Pupils were dilated with one drop of 1.0% Tropicamide eye drops 10 mg/ml (Alcon Laboratories) and lubricant eye drops (Refresh Tears Plus, Allergan, USA) were used throughout the procedure to maintain corneal moisture and clarity. OCT images were obtained in mice using a commercially available scanning laser ophthalmoscope, the Heidelberg Retina Angiograph 2 (HRA 2; Heidelberg Engineering, Germany). The imaging system was adapted for the optics of the mouse eye with a 55° wide-angle lens.

### Histology

Eyes were collected at various times and fixed with 2% paraformaldehyde for at least 24 hours. Samples were embedded in paraffin and 10 µm sections were prepared for haematoxylin and eosin staining using standard methods. Immunofluorescent studies were performed on 10 µm frozen sections. HEL was detected using polyclonal rabbit antibody (United States Biological, USA).

### Disease Scoring

Disease was assessed *in vivo* using optical coherence tomography (OCT) and in histological H&E stained sections of the eye. Severity of disease was scored on OCT as inflammatory cell infiltrate within the vitreous (vitritis) using a four-point scale (0 = no vitritis; 1 = mild; 2 = moderate; 3 = severe). Histological severity of EAU was graded using a previously reported grading system^66^.

### Flow cytometry

Spleen and lymph node cells were prepared by passage through a stainless steel sieve. Erythrocytes were lysed as above, then washed thoroughly with MOBS (mouse osmolarity buffered saline)/0.5%FCS. Eyes were dissected to separate the anterior segment from the posterior. The anterior segment was dissected further to yield the iris/ciliary body tissues while the posterior segment was dissected to separate the retina from the choroid/RPE tissue. Retinas from both globes were pooled for each mouse. The iris and choroid/RPE tissue samples were pooled from multiple mice (up to five/group) before all tissue was minced and digested in a mixture of 10 µg/ml Liberase TM (Roche, Germany) and 10 µg/ml DNAse I (Sigma, USA) in PBS for 40 minutes. Antibodies used for analysis (CD3, TCRβ, CD4, CD8, CD45.1, CD45.2, Foxp3, IFN-γ, IL-17) were purchased from BD Biosciences (USA) and Biolegend (USA). Expression of the transgenic TCR was detected using the MAb 1G12, biotinylated anti-mouse IgG1 (BD Biosciences), and a streptavidin conjugated PE or APC (BD Biosciences). Dead cells were detected using propidium iodide (PI) or FVS 620 (BD Biosciences), and at least 100000 live events were collected for analysis. Cells were collected using a FACSCanto® or a Fortessa® (Becton Dickinson, USA) and analysed using FlowJo Analysis Software (TreeStar, USA).

### Statistical analyses

Statistical differences were analysed with InStat® Prism software (GraphPad Software Inc, USA). Mann-Whitney non-parametric testing was used for all comparisons between 2 groups and for the analyses shown in Fig. 2C. One-way analysis of variance (ANOVA) with two-tailed, Students *t*-test *post hoc* analyses was used for all comparisons involving more than 2 groups.

### Data Availability

All data generated or analysed during this study are included in this published article (additional data are available from the corresponding author on reasonable request).
